# Supplementary figures and images for: Absence of the Fragile X messenger ribonucleoprotein alters response patterns to sounds in the auditory midbrain
Source: Front Neurosci. 2022 Sep 16;16:987939. doi: 10.3389/fnins.2022.987939 (PMC9523263; doi:10.3389/fnins.2022.987939)

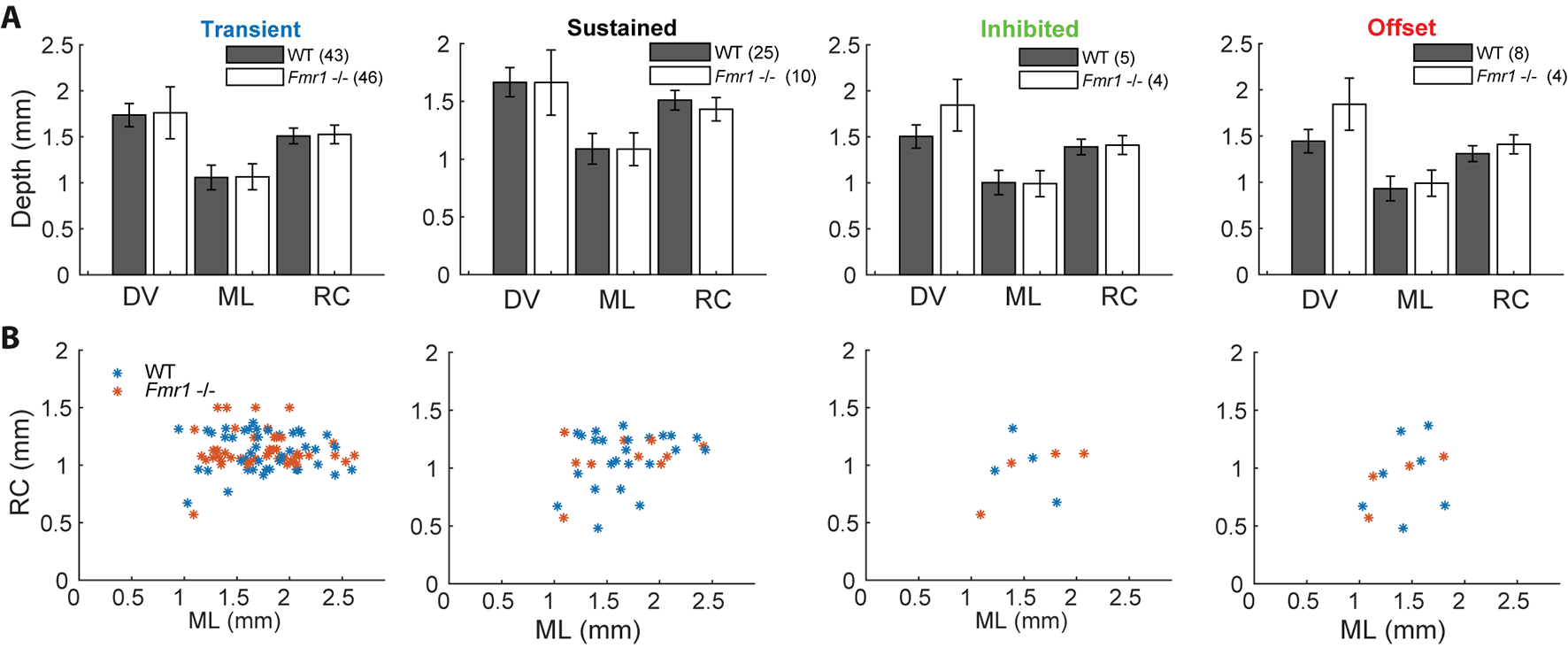

Supplement: Supplementary Figure 1 — Detailed stereotactic recording locations in both genotypes. (A) Quantification of recording stereotactic depths in respectively: (left) transient neurons, (middle left) sustained neurons, (middle right) inhibited neurons, (right) offset neurons. (B) Quantifications of the respective Rostro-Caudal to Medio-Lateral stereotactic recording locations in resp.: (left) transient neurons, (middle left) sustained neurons, (middle right) inhibited neurons, (right) offset neurons. [file Image_1.tif]

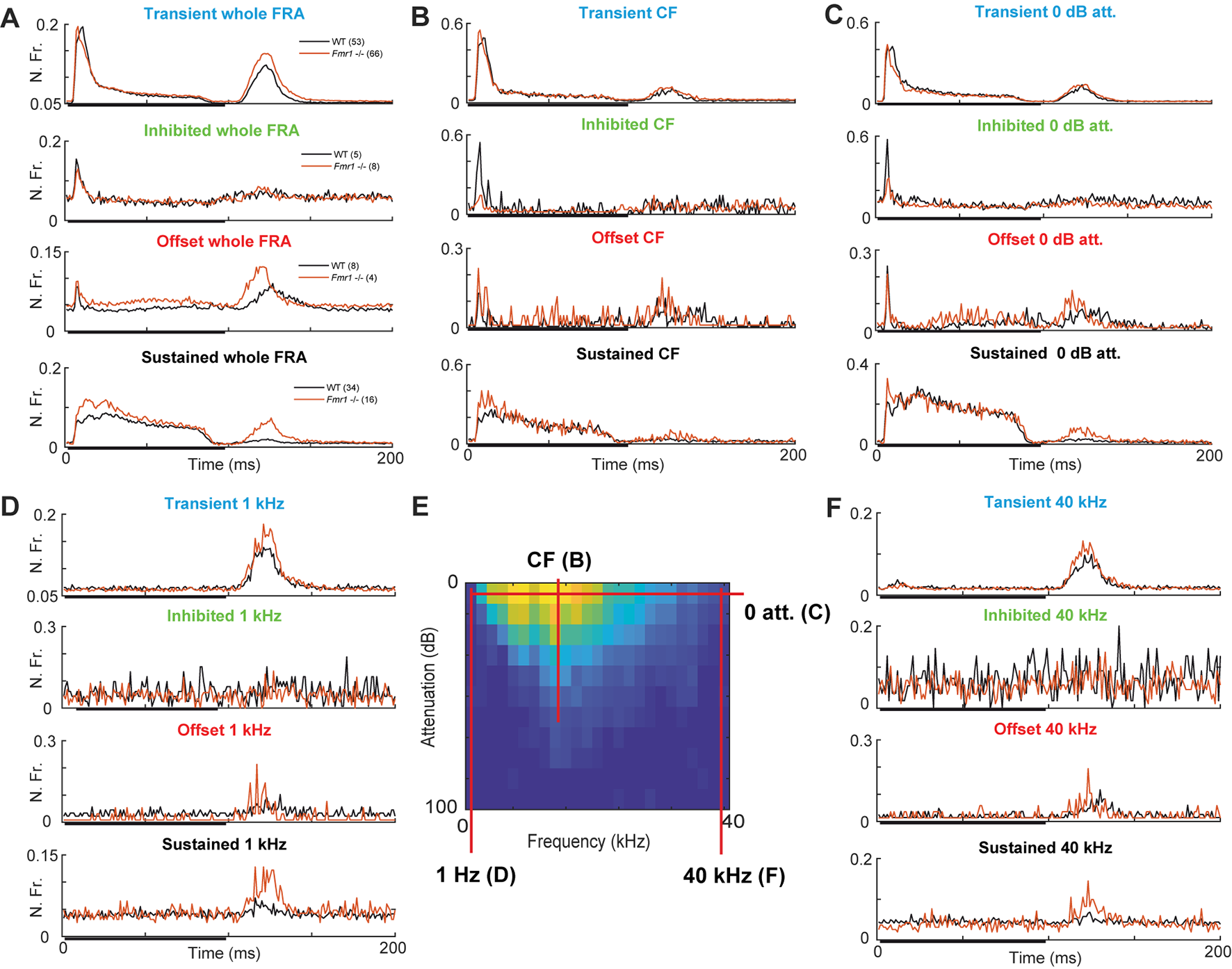

Supplement: Supplementary Figure 2 — Localized estimates of the different firing patterns exhibited in defined sub-sections of the FRA. (A) Averaged compound PSTH of all conditions in the FRA for respectively: (above) all transient WT and all Fmr1 -/- neurons, (middle above) all inhibited WT and all Fmr1 -/- neurons, (middle below) all offset WT and all Fmr1 -/- neurons, (below) all sustained WT and all Fmr1 -/- neurons. (B) Averaged PSTH at the CF for all intensity above threshold for respectively: (above) transient WT and Fmr1 -/-, (middle above) inhibited WT and Fmr1 -/-, (middle below) offset WT and Fmr1 -/-, (below) sustained WT and Fmr1 -/-. (C) Averaged PSTH of 0 dB attenuation for all frequencies for respectively: (above) transient WT and Fmr1 -/-, (middle above) inhibited WT and Fmr1 -/-, (middle below) offset WT and Fmr1 -/-, (below) sustained WT and Fmr1 -/-. (D) Averaged PSTH of 1 kHz pure tone stimulations at all intensities for respectively: (above) transient WT and Fmr1 -/-, (middle above) inhibited WT and Fmr1 -/-, (middle below) offset WT and Fmr1 -/-, (below) sustained WT and Fmr1 -/-. (E) Schematic overview of a FRA with red highlights the different sub-section chosen for the illustrated PSTH. (F) Averaged PSTH of 40 kHz pure tone stimulation for all intensities for respectively: (above) transient WT and Fmr1 -/-, (middle above) inhibited WT and Fmr1 -/-, (middle below) offset WT and Fmr1 -/-, (below) sustained WT and Fmr1 -/-. [file Image_2.tif]

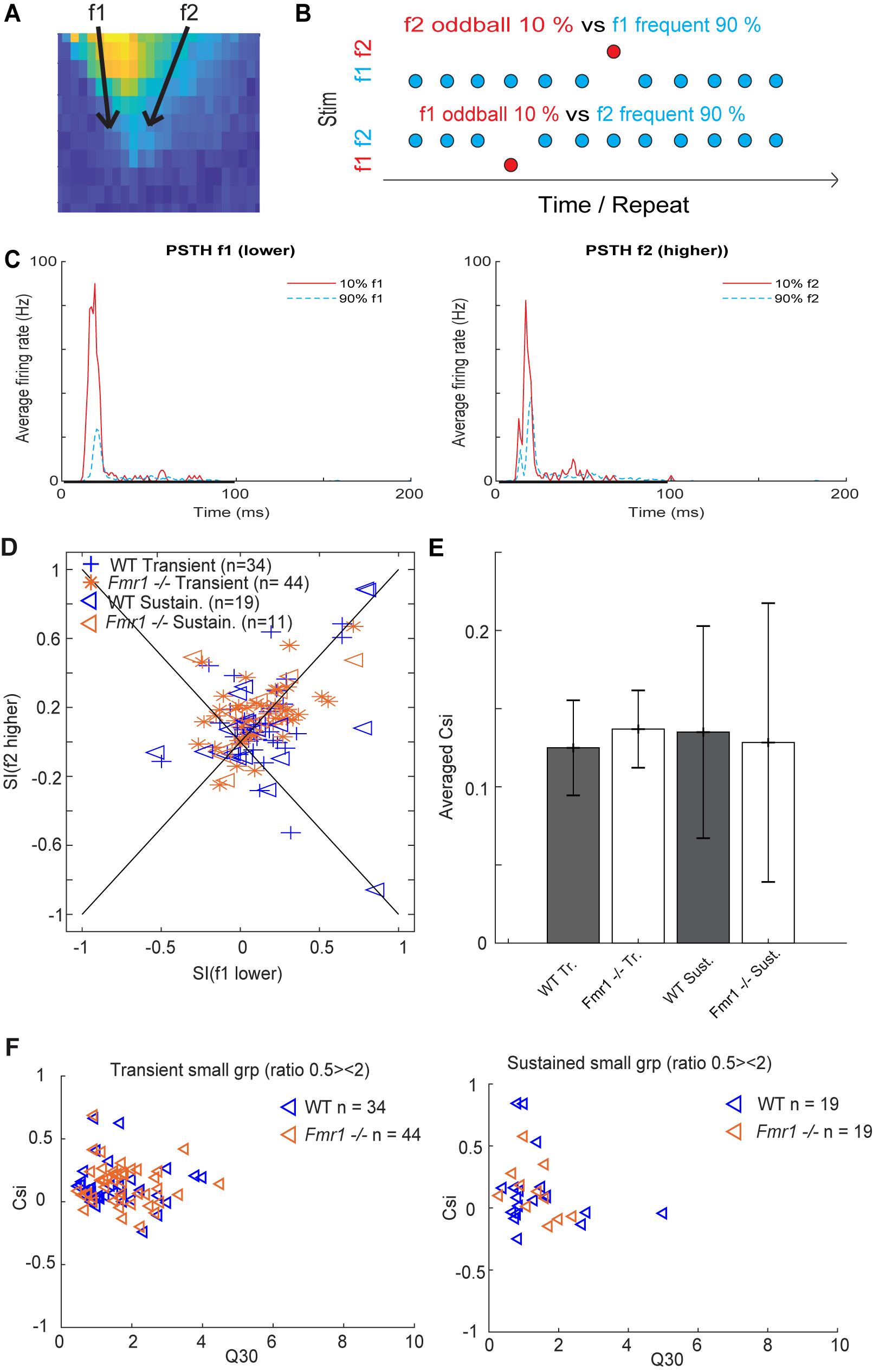

Supplement: Supplementary Figure 3 — Neurons in the IC exhibit poor stimulus-specific adaptaion. (A) Schematic FRA and the location around the CF chosen to identify f1 and f2. (B) Representation of (blue and red) the two frequencies of stimulation with the repeats used to bot oddball protocols. (C) PSTH to the different conditions in an adaptive neuron, (left) response to a (red) f1 odd, (blue) f1 frequent stimulation; (right) responses to an (red) f1 odd, (dashed-blue) f2 frequent stimulations. (D) Quantifications of the SSA to a standard 90/10% oddball paradigm represented according to each neuron’s adaptive responses to both the lower frequency (f1-x-axis) and the higher frequency (f2-y-axis). (E) Averaged adaptation strength to both frequencies quantified by the common SSA index (CSI). F Individual CSI plotted against their respective Q30 in both WT and Fmr1 -/- Transient neurons (left) and Sustained neurons (right). (F) Illustrations of the tuning width versus the CSI values for (left) transient neurons versus (right) Fmr1 -/- neurons. [file Image_3.tif]
